# Coping as a resource to allow for psychosocial adjustment in fatal disease: results from patients with amyotrophic lateral sclerosis

**DOI:** 10.3389/fpsyg.2024.1361767

**Published:** 2024-04-04

**Authors:** Julia Finsel, Angela Rosenbohm, Raphael S. Peter, Hansjörg Bäzner, Axel Börtlein, Silke Dempewolf, Martin Schabet, Martin Hecht, Andreas Kohler, Christian Opherk, Andrea Nägele, Norbert Sommer, Alfred Lindner, Dietrich Rothenbacher, Albert C. Ludolph, Gabriele Nagel, Dorothée E. Lulé

**Affiliations:** ^1^Department of Neurology, Ulm University, Ulm, Germany; ^2^Institute for Epidemiology and Medical Biometry, Ulm University, Ulm, Germany; ^3^Department of Neurology, Katharinenhospital Stuttgart, Stuttgart, Germany; ^4^Department of Neurology, RKH Klinikum Ludwigsburg, Ludwigsburg, Germany; ^5^Department of Neurology, Klinikum Kaufbeuren, Kliniken Oberallgäu-Kaufbeuren, Kaufbeuren, Germany; ^6^Department of Neurology, Klinikum am Gesundbrunnen Heilbronn, Heilbronn, Germany; ^7^Department of Neurology, Christophsbad Göppingen, Göppingen, Germany; ^8^Department of Neurology, Marienhospital Stuttgart, Stuttgart, Germany; ^9^German Center for Neurodegenerative Diseases (DZNE), Ulm Site, Ulm, Germany

**Keywords:** amyotrophic lateral sclerosis, coping, psychosocial adaptation, quality of life, depressiveness, wellbeing

## Abstract

**Background:**

Amyotrophic lateral sclerosis (ALS) is a fatal disorder, which imposes a severe emotional burden on patients. Appropriate coping mechanisms may alleviate this burden and facilitate wellbeing, with social support known to be a successful coping strategy. This observational study aimed to determine the interplay of general coping traits of hope for success and fear of failure, coping behavior of social activity, and patients' wellbeing.

**Methods:**

In this cross-sectional study, patients with ALS from a clinical-epidemiological registry in Southwestern Germany were interviewed regarding coping traits (achievement-motivated behavior: hope for success and fear of failure), coping behavior of social activity, and psychosocial adjustment, determined using measures of depressiveness, anxiety [both measured by Hospital Anxiety and Depression Scale (HADS)], and quality of life [Anamnestic Comparative Self-Assessment (ACSA)]. Demographics, clinical [ALS Functional Rating Scale revised version (ALSFRS-R)], and survival data were recorded.

**Results:**

A total of 868 patients [60.70% male patients, mean age: 64.70 (±10.83) years, mean ALSFRS-R: 37.36 ± 7.07] were interviewed. Anxiety in patients was found to be associated with a high fear of failure. In contrast, a generally positive attitude in patients exemplified in high hopes for success was associated with better wellbeing. Finally, coping behavior of social activity explained up to 65% of the variance of depressiveness among the patients with ALS.

**Conclusion:**

In this study, we present evidence that the wellbeing of patients with ALS is not an immediate fatalistic consequence of physical degradation but rather determined by coping traits and behavior, which may be trained to substantially increase the wellbeing of patients with ALS.

## 1 Introduction

Amyotrophic lateral sclerosis (ALS) is the most frequent adult-onset motor neuron disease with typical onset after 60 years of age, characterized by progressive physical, respiratory, and swallowing impairments. The median survival after disease onset ranges from 2 to 4 years (Chiò et al., 2009). During the disease course, patients have to deal with severe restrictions in daily life, intensive use of medical services, and physical dependency on others (Oliver et al., 2016), resulting in a tremendous emotional burden (McLeod and Clarke, 2007). Patients often experience heightened levels of depressiveness and anxiety in the initial phase of ALS and shortly after diagnosis (Kurt et al., 2007; Vázquez Medrano et al., 2020). However, a majority of patients may gain satisfactory wellbeing in the long course (Rabkin et al., 2000), despite increasing physical disability, even when reaching the locked-in state (Kuzma-Kozakiewicz et al., 2019). This aspect indicates that patients develop highly individualized coping strategies to preserve their psychological wellbeing.

This coping process depends on several intrinsic and extrinsic factors, of which the physical state is only one among many (Nelson et al., 2003). In general, coping can be categorized into coping strategies and coping styles (Lazarus and Folkman, 1984). Coping strategies involve distinct behavioral actions during a patient's stress episode (Lazarus and Folkman, 1984; Rabari et al., 2023). Most studies regarding coping in ALS focus on coping strategies (Matuz et al., 2010, 2015; Montel et al., 2012; Tramonti et al., 2012; Jakobsson Larsson et al., 2016; Leandro et al., 2022), with “seeking social support” being the primary coping strategy in the sense of efforts to seek information, emotional support, or instrumental support (Matuz et al., 2010; Leandro et al., 2022). Coping strategies are plentiful, but it has been theorized that individuals usually react to stressful situations in a certain way, based on a specific coping style (Roth and Cohen, 1986). Coping styles, or traits, are related to general personality traits and the result of previous experience (Lazarus and Folkman, 1984). Additionally, these styles are usually divided into the tendencies of approach and avoidance (Roth and Cohen, 1986). However, limited knowledge exists regarding coping traits according to personality traits in ALS, apart from an elevated anxiety trait being associated with decreased quality of life (QoL) (Siciliano et al., 2019). The specific personality traits of patients with ALS suggest a strong desire to excel in tasks and a high aim for achievement, compared to healthy controls (Parkin Kullmann et al., 2018). However, the extent to which coping traits affect the wellbeing of patients with ALS remains extensively unexplored, prompting us to address this gap.

A concept used to quantify these coping traits is the achievement-motivated behavior (AMB) (Atkinson, 1957). AMB reflects the general tendency of individuals to evaluate their behavior in situations where they perceive the potential to improve the outcomes (Atkinson, 1957). Achievement motive splits into approach (hope of success) and avoidance (fear of failure) tendencies (Atkinson, 1957; Heckhausen, 1963, 1991). The difference between hope of success and fear of failure serves as a predictor for the overall strength of individual's motives for achievements. These motives are regarded as a personality trait and are triggered by social incentives, such as external expectations (Atkinson, 1957). In ALS, the debilitating condition limits options to fulfill external expectations (e.g., lack of gratification in a job as most have to retire following the diagnosis), resulting in patient's mastery is primarily driven by their inner achievement motive. However, there is limited knowledge on how this inner achievement motive, as an indicator of coping traits, interacts with the successful psychosocial adaptation of patients affected by ALS.

The objective of this study was to characterize the potentially protective effect of general coping traits and current social behavior on the wellbeing of patients with ALS. To exclude selection bias stemming from highly motivated patients, we included a large cross-sectional sample of patients from the ALS registry Swabia in Southwestern Germany (Nagel et al., 2013), which is now among the most prominent European ALS registries and covers a majority (82%) of patients in this area.

## 2 Methods

### 2.1 Study design and procedures

This study is conducted as a cross-sectional and observational investigation and is part of the ALS registry Swabia in Southwestern Germany (Nagel et al., 2013), a clinical-epidemiological registry established in October 2010. Total ethical approvals were obtained from the ethical committees of the University of Ulm (No. 11/10), the medical association of the state of “Baden-Wuerttemberg” (Landesärztekammer, No. B-F-2010-062), and the medical association of the state of “Bayern” (No. 7/11300). All participants gave verbal and written informed consent prior to inclusion in the study. The study protocol with recruitment procedures, inclusion criteria, and details on the standardized data collection sheet have been previously published (Nagel et al., 2013; Rosenbohm et al., 2017). The relevant measures for this particular study are reported in detail under the Measures section. All patients underwent a neurological examination conducted by the collaborating neurologists. Additional clinical information and neuropsychological data were collected by neuropsychologists and trained study nurses using the standardized data collection sheet (Nagel et al., 2013). In a case-control study, healthy controls were interviewed who were matched for age, living area, and sex, for further details please see Nagel et al. (2013).

### 2.2 Participants' characteristics

A total of 868 patients with ALS were interviewed (Table 1) (Nagel et al., 2013; Rosenbohm et al., 2017). The diagnosis was verified and classified according to the revised El Escorial criteria (Brooks et al., 2000; Ludolph et al., 2015). The mean age at the time of interview was 64.70 (± 10.83) years. The physical function was determined using the ALS functional rating scale revised version (ALSFRS-R) (Cedarbaum et al., 1999), ranging from 0 (no residual volitional motor control) to 48 points (full motor control). Patients exhibited moderate physical impairments (ALSFRS-R mean = 37.36 ± 7.07) with a mean bulbar involvement of 9.84 (±2.76; max. 12), a mean spinal involvement of 16.66 (±5.37; max. 24), and a mean respiratory involvement of 10.81 (±2.03; max. 12). Mean disease duration from symptom onset to interview was 14.68 (±11.44) months, indicating an early phase of the disease course. The progression rate (calculated as 48 minus current ALSFRS-R divided by months since symptom onset) ranged from moderate to fast (Kollewe et al., 2008) (mean = 1.07 loss of ALSFRS-R score per month ±1.13; Table 1). Patients' vital status was annually verified through record linkage with the central registration database and requests in the regional registration offices of Baden Württemberg and Bavaria. The censoring date for the survival analyses was October 30, 2021. In total, 604 (69%) died with the mean survival rate of 32.74 (±18.14) months after onset. A total of 788 healthy controls were interviewed in the case-control study (Nagel et al., 2013).

**Table 1:** Characteristics of ALS cases and healthy case controls.

	**Patients (*****N*** = **868)**		**Case controls (*****N*** = **788)**		**Statistics**		
	**Mean/*N***	**SD/%**	**Mean/*N***	**SD/%**	***F*/*U*/χ^2^**	***p***	**η^2^**
**Age**	64.70	10.83	64.10	10.34	0.75	0.39	0.001
**Sex**							
Male	527	60.7%	479	60.8%			
Female	341	39.3%	309	39.2%			
**Months since onset**	14.68	11.44					
**Progression rate**	1.07	1.13					
**Survival in months**	32.74	18.14					
**ALSFRS-R total**	37.36	7.07					
ALSFRS-R bulbar	9.84	2.76					
ALSFRS-R spinal	16.66	5.37					
ALSFRS-R respiratory	10.81	2.03					
**School years**	9.65	1.93	10.04	2.12	8.51	0.004	0.009
**University years**	4.09	2.39	4.88	2.11	5.29	0.023	0.030
**Partnership**	862		786		14.54	0.002	0.031
Single	64	7.4%	35	4.5%			
Married	660	76.6%	633	80.5%			
Divorced	75	9.0%	44	5.6%			
Widowed	63	7.3%	74	9.4%			
**Children**	860		780		0.94	0.35	0.024
Yes	709	82.4%	657	84.2%			
No	151	17.6%	123	15.8%			
**Living condition**	618		181		14.93	0.002	0.089
Alone	106	17.2%	15	8.3%			
Family	497	80.4%	165	91.2%			
Nursing home	15	2.4%	0	0%			
Shared flat	0	0%	1	0.6%			
**Cognition**	576		377				
ECAS total	102.14	17.74	107.35	13.74	89,753.00	< 0.001	0.028
ECAS memory	14.84	5.22	16.66	4.58	85,042.50	< 0.001	0.034
ECAS visuospatial	11.28	1.36	11.80	0.81	81,652.50	< 0.001	0.044
ECAS language	25.20	3.55	26.86	2.27	67,401.00	< 0.001	0.067
ECAS verbal fluency	15.89	5.52	14.33	5.44	127,779.00	< 0.001	0.030
ECAS executive function	34.79	7.61	37.68	5.66	81,733.50	< 0.001	0.042

ALSFRS-R, ALS functional rating scale revised version; ECAS, Edinburgh Cognitive and Behavioral ALS Screen; N, number; SD, standard deviation.

### 2.3 Measures

#### 2.3.1 Cognition

The Edinburgh Cognitive and Behavioral ALS Screen (ECAS) (Abrahams et al., 2014; Lulé et al., 2015) is a specifically designed cognitive and behavioral instrument for patients with ALS to examine language, verbal fluency, executive functioning, memory, and visuospatial functioning. The concurrent validity of the German version with similar instruments is moderate to high (*r* = 0.46–0.58), showing high specificity (75%−100%), medium sensitivity (33%−50%), and moderate to high inter-rater reliability (Cohen's kappa value = 0.4–0.9) depending on the cognitive domain (Lulé et al., 2015).

A total of 576 patients were physically capable of completing the ECAS, while 377 controls were tested with the ECAS. Additionally, behavioral data were collected for 523 patients with interviews of caregivers (all first-degree relatives).

#### 2.3.2 Coping traits

Coping traits regarding hope for success and fear of failure were determined using the short version of the Achievement Motive Scale (AMS) (Gjesme and Nygard, 1970; Lang and Fries, 2006), which consists of a 10-item scale with respective ratings ranging from 4 = strongly agree to 1 = strongly disagree. The achievement motive is determined according to either the component of hope for success (five items) or fear of failure (five items). The difference between both scales serves as a measure of the individual level of achievement motive. The reported internal consistency is Cronbach's alpha = 0.71–0.86, and the validity coefficient ranges between 0.51 and 0.79 (Lang and Fries, 2006).

#### 2.3.3 Wellbeing

Wellbeing was determined by measures of affective state (depressiveness and anxiety) and quality of life (QoL). Depressiveness and anxiety were examined using the self-reporting Hospital Anxiety and Depression Scale (HADS) (Herrmann-Lingen et al., 2011), with each scored between 0 and 21. Scores ranging between 11 and 21 indicated probable cases of clinically significant depressiveness and anxiety, respectively. Cronbach's alpha is reported as 0.67 to 0.93, sensitivity and specificity are both at 80%, and correlation with similar instruments is reported to be between 0.48 and 0.83 (Bjelland et al., 2002).

QoL was assessed with the Anamnestic Comparative Self-Assessment (ACSA) (Bernheim and Buyse, 1983; Bernheim, 1999). ACSA is a single-item instrument developed in clinical settings to assess subjective wellbeing. Participants were asked to define the periods of their best and worst QoL throughout their lifetime and to rate their current level (−5 = as bad as possible to +5 = as good as possible) according to the anchors of best and worst periods. Correlations with conventional happiness questions have been reported to range between 0.43 and 0.67 (Bernheim et al., 2006; Verhofstadt et al., 2019), while correlation with a multi-item life satisfaction instrument was reported as 0.59 (Bernheim et al., 2006). In patients with cancer, concurrent validity was further suggested by significant correlations between ACSA and the gravity of the disease, physical performance status, and response to treatment (Bernheim and Buyse, 1983).

#### 2.3.4 Current behavior and demographics

Current behavior regarding social activity was defined according to the frequency of activities with family members and close friends (1 = less than once a month to 5 = on a daily base). Demographics such as the living situation (alone/with partner or family/others), partnership (single/married or partner/divorced/widowed), and the number of children were taken into consideration. Furthermore, participation in voluntary or club activities (yes/no) and hours dedicated to such activities were recorded. Club membership in Germany is the membership in an organization of society with specific goals, including clubs of physical activities, but also clubs of traditions (civic and home associations), clubs of specific hobbies (gardening, philately, animal breeding, etc.), clubs of music, clubs of environment and nature conservation, clubs for support groups (unemployment, alcoholism, patients with specific diseases or their caregivers, etc.), clubs of charity, clubs of worldview, and clubs of that particular in fundraising (for kindergartens, schools, theaters, individual education, etc.) (Müller-Jentsch, 2008).

### 2.4 Statistical analysis

Statistical analyses were conducted using IBM^®^ SPSS version 25.0. All quantitative, continuous data are given as mean and SD. Categorical data are described as absolute frequencies and ratios. Due to the large sample size of *N* > 100 participants, normality distribution can be assumed (Lumley et al., 2002), and ANOVA, being robust to possible violations of non-normality (Blanca et al., 2017), was used to compare patients with controls for coping traits, current coping behavior, demographics (age, school years, years of university studies), and wellbeing. According to the Levene test, homogeneity of variances was present for all variables except for some subscores of the ECAS, so group comparisons for cognitive status were conducted using the Mann–Whitney-*U* tests. The chi-square test was conducted to compare partnership, number of children, and living conditions. Regression models and curve fit were conducted with independent variables for general coping traits of achievement motives (hope for success, fear of failure, total score) and current coping behavior (activity in voluntary work, club membership and with family members, demographics); dependent variable was defined as wellbeing (anxiety and depressiveness measured with HADS, subjective measure of global of life with ACSA). Due to large sample size, only predictors with *p* < 0.001 are considered significant. Regarding effect sizes, η^2^ = 0.01 indicates a small effect, η^2^ = 0.06 indicates a medium effect, and η^2^ = 0.14 indicates a large effect. All statistical tests, apart from the regression model, were two-sided, and the significance level was set at *p* = 0.05.

## 3 Results

### 3.1 Demographics

Compared to healthy controls, patients with ALS showed no significant differences with regard to demographics, apart from fewer university years, being single with a small effect size, and having more patients living alone with a medium effect size (Table 1).

### 3.2 Cognition

A total of 90 (15.6%) patients exhibited mild cognitive impairments according to age and education-adjusted cut-offs (Loose et al., 2016). A total of 26 (6.9%) controls were found to have cognitive impairments according to the cut-offs mentioned above. Patients differed significantly from controls in all ECAS subscores (Table 1).

Apathy (*N* = 131, 25%) was the most common behavioral change in patients, followed by loss of social interest (*N* = 73, 14%), loss of compassion (*N* = 56, 11%), hyperorality (*N* = 40, 8%), stereotyped behavior (*N* = 28, 5%), which were subsumed under five behavioral domains (disinhibition, apathy, loss of sympathy, stereotyped behavior, and hyperorality); however, none of the participants fulfilled criteria of behavioral variant FTD according to Strong et al. (2017).

### 3.3 Wellbeing and coping

Patients with ALS showed reduced wellbeing, as evidenced by significantly higher anxiety and depressiveness scores (incidence of clinically relevant depression 4.6 vs. 28.2%, and anxiety 4.4 vs. 24.5%) and a lower subjective QoL. For both groups, wellbeing was in a neutral to positive range (mean 0.19 ± 2.37 vs. mean 2.34 ± 1.59; Table 2).

**Table 2:** Comparison of patients with ALS and healthy case-controls on general traits, wellbeing and current behavior.

	**Patients**			**Case controls**			**Statistics**		
	***N***	**Mean/%**	**SD**	***N***	**Mean/%**	**SD**	***F*/χ^2^**	***p***	***η^2^***
**ACSA (sQoL)**	817	0.19	2.37	674	2.34	1.59	407.45	< 0.001	0.215
**HADS**	832			781					
Anxiety		7.40	4.31		3.93	3.26	326.07	< 0.001	0.171
Depressiveness		8.77	4.70		3.42	3.11	718.15	< 0.001	0.308
**AMS**	868			788					
Hope for success		15.27	3.41		15.53	2.84	2.83	0.09	0.002
Fear of failure		10.46	3.89		10.17	3.27	2.68	0.10	0.002
Achievement motive		4.83	5.45		5.36	4.55	4.39	0.04	0.003
**Current behavior**									
**Voluntary activity (VA)**	618			179			40.37	< 0.001	0.225
Yes	104	16.8%		70	39.1%				
No	514	83.2%		109	60.9%				
**Club membership (CM)**	617			182			44.29	< 0.001	0.235
Yes	126	20.4%		82	45.1%				
No	491	79.6%		100	54.9%				
**Hours for VA**	95	9.85	13.37	67	11.57	10.96	0.75	0.39	0.005
**Hours for CM**	111	10.83	12.84	76	11.87	8.76	0.38	0.54	0.002
**Activity with family**	618			180			27.36	< 0.001	0.166
Never	76	12.3%		2	1.1%				
< 1 × /month	105	17.0%		28	15.6%				
1 × /month	149	24.1%		38	21.1%				
1 × /2 weeks	91	14.7%		28	15.6%				
1 × /week	189	30.6%		80	44.4%				
Every day	8	1.3%		4	2.2%				

AMS, Achievement motive scale (short version); ACSA, Anamnestic comparative self-assessment; sQoL, subjective quality of life; HADS, Hospital Anxiety and Depression Scale; N, number; SD, standard deviation.

Regarding coping traits, no significant group differences were found for the total achievement motive score, hope for success, or fear of failure.

Current coping behavior with regard to social activity was significantly different since patients spent less time with their families. Patients reported engaging in fewer social activities such as a club member or doing voluntary activity; the hourly effort for these activities was similar between both groups (Table 2).

### 3.4 Impact of general coping traits and current behavior on wellbeing

**Figure 1:**
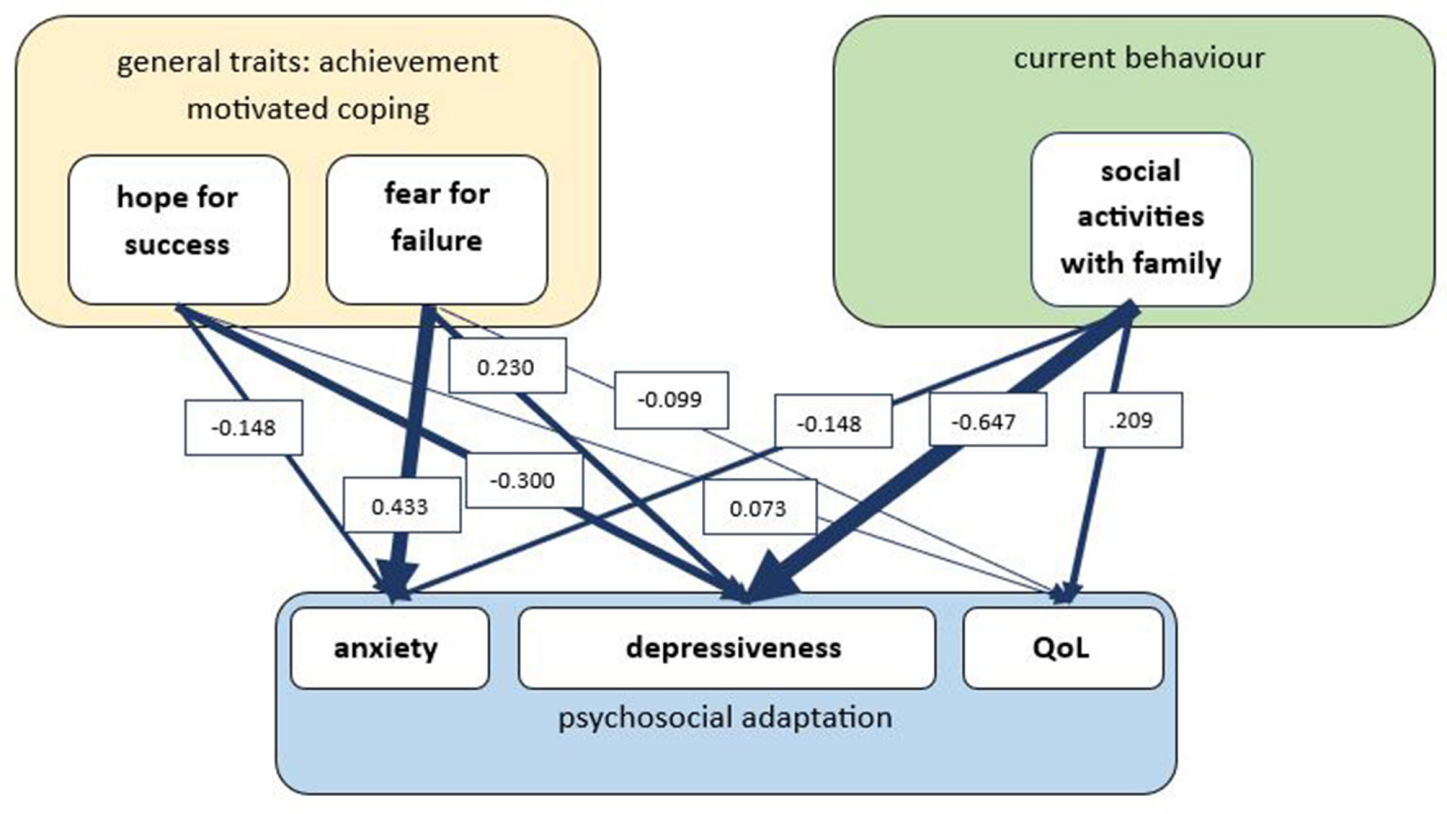
Prediction model of psychosocial adaptation. General traits of achievement motives are defined by hope of success and fear of failure (measured with Achievement Motive Scale, AMS); current behavior is defined by social activities with family members; psychosocial adaptation is defined by anxiety and depressiveness (measured with Hospital Anxiety and Depression Scale, HADS) and subjective quality of life (measured with Amnestic Comparative Self-Assessment, ACSA). Regression analysis with B-values indicating explained variance; due to large sample size, only predictors with p < 0.001 are considered significant.

The prediction model of psychosocial adaptation can be found in Figure 1. The most substantial effect was observed for anxiety (*R*^2^ = 0.173, *F* = 38.85, *p* < 0.001), which was predicted by fear of failure (*B* = 0.433 *p* < 0.001), explaining 43% of the variance, and hope for success, explaining 15% of the variance (*B* = 0.148, *p* = 0.007). Depressiveness was also predicted by coping traits of hope for success, explaining 30% of the variance (*R*^2^ = 0.146, *F* = 34.107, *p* < 0.001; *B* = −0.300, *p* < 0.001), and fear of failure accounting for 23% of the variance (*B* = 0.230, *p* < 0.001). Additionally, depressiveness was predicted by current coping behaviors of social activity (*B* = −0.647, *p* < 0.001), explaining 65% of the variance. Finally, QoL was determined by coping traits (*R*^2^ = 0.062, *F* = 12.252, *p* < 0.001), hope for success explained 7% (*B* = 0.073, *p* = 0.015) and fear of failure explained 10% (*B* = −0.099, *p* < 0.001). The current behavior of family activities explained 21% of the variance in QoL (*B* = 0.209, *p* = 0.004). A summary of the relationships between coping traits and coping behavior with wellbeing is shown in Table 3.

**Table 3:** Effect of coping traits and coping behavior on wellbeing.

	**Coping traits**		**Coping behavior**
	**Hope for success**	**Fear of failure**	**Social activities**
Anxiety	↓	↑*↑↑*	↓
Depressiveness	↓↓	↑↑	↓*↓↓*
QoL	↑	↓	↑↑

“↑” indicates a positive relationship, “↓” indicates a negative relationship; B-value of 0–0.20 = small variance explained by variable indicated by “↑/↓,” 0.21–0.40 medium variance indicated by “↑↑/↓↓,” and B-value >0.40 high variance explained indicated by “↑*↑↑*/↓*↓↓*”.

QoL, quality of life.

None of the other factors (demographics, voluntary/club activity) were found to be significant predictors of wellbeing.

## 4 Discussion

The Southwestern German ALS registry served as a platform for exploring the potentially protective effect of general coping traits and current social behavior on the wellbeing of patients with ALS. We present evidence that coping traits and behavior essentially predict psychosocial adaptation and, thus, wellbeing of patients with ALS. Subsequently, general traits of achievement motives predicted wellbeing similar to current behaviors of social participation. This study expands the current evidence on coping with ALS, which was primarily focused on researching coping strategies rather than traits (Matuz et al., 2010), and adds achievement motives as a potential element of the coping process to preserve wellbeing despite ALS. Furthermore, our results are in line with the model of coping proposed by Lazarus and Folkman (1984), wherein achievement motives as part of personality traits may influence of selection and implementation of coping behaviors (Lazarus and Folkman, 1984; Connor-Smith and Flachsbart, 2007). The link between personality and implemented coping strategies while dealing with severe diseases has also been demonstrated recently in patients with cancer (Rabari et al., 2023); however, the relationship between personality traits and coping strategies appears to vary across different population samples (Connor-Smith and Flachsbart, 2007).

The study provides evidence that the coping trait of hope for success has a positive effect on the wellbeing of patients with ALS, as measured by affective state and quality of life. Conversely, the effect of fear of failure is linked with increased anxiety and depressiveness. ALS is inherently associated with several losses in different contextual situations, which may not only include the loss of their physical strength and autonomy and possible dependence on the assistance of others for daily care and on medical devices for survival (The EFNS Task Force on Diagnosis and Management of Amyotrophic Lateral Sclerosis et al., 2012; Lulé et al., 2018) but may also include loss of the employment, leading to financial security and professional expression. Eventually, patients with ALS often confront the fact that they will be losing life itself within a short time frame. The patients who have always been anxious and afraid of losing, expressed in fear of failure, may suffer even more in the face of the multiple changes imposed on them in the context of ALS. Patients with a high fear of failure may feel less sense of control and may tend to use less problem-focused strategies. However, understanding of control and problem-focused strategies are both correlated with better wellbeing of patients with ALS (King et al., 2009; Matuz et al., 2015; Jakobsson Larsson et al., 2016). On the other hand, the patients who have a positive attitude, as expressed in hope for success, find it easier to cope with the devastating condition according the hereby presented data. This aspect aligns with earlier findings, wherein patients with ALS who exhibit positive action and positive thinking tend to experience fewer depressive symptoms (Jakobsson Larsson et al., 2016).

Our study revealed a general lower wellbeing of patients with ALS compared to controls, characterized by lower subjective QoL and higher rates of anxiety and depressiveness, highlighting the negative effects of the disease. Shortly after diagnosis, many patients show a reactive episode of low wellbeing, as indicated by increased depressiveness and low QoL (Vázquez Medrano et al., 2020). Our study population was in rather early disease stage. Additionally, the study population exhibited a rather high progression rate, which is known to be associated with low wellbeing (Lulé et al., 2013). However, psychosocial adaptation is possible in the course of ALS, despite the severity of the medical condition and the emotional burden it imposes. This phenomenon is a general one observed in severe diseases with different etiologies, subsumed under the wellbeing paradox (Herschbach, 2002). However, this adaptation is only achievable if certain prerequisites are fulfilled. According to the data presented, these prerequisites include extrinsic (current coping behavior, here measured with social activity) and intrinsic factors related to general coping traits. While we found no differences in general coping traits between patients with ALS and controls, patients reported less social activity compared to controls. This is unfortunate, since coping strategies of “seeking social support” have been shown to be the most successful coping strategy in ALS (Matuz et al., 2010, 2015; Tramonti et al., 2012; Leandro et al., 2022) and family members were named to be most helpful regarding coping (Hecht et al., 2002). Our data support this concept to be valid: social activities with family members explained up to 65% of the variance in depressiveness in patients with ALS and to a lesser degree of quality of life and anxiety. This effect can be easily modulated by families and friends and yielded by palliative care teams by providing social inclusion and participation of patients in social activities. Especially for those patients living in nursing homes, where home residents are most of their time unoccupied and with little interaction (Hoel et al., 2022), social activities, the quality of life may be endangered. Thus, it is of utmost importance to monitor the wellbeing and coping of patients with ALS from early on and during the course of the disease.

### 4.1 Limitations

This work presents no causality but correlations. Therefore, causal conclusions about the relationship between coping traits, coping behaviors and wellbeing of patients with ALS cannot be drawn. Patients who are depressed may as well be the ones with reduced social activities. Additionally, the affective state could be modulating the tendency for hope for success or fear of failure. Longitudinal studies are needed to strengthen the evidence for the proposed relationships. To better understand the relationship between the achievement motives and actual coping strategies used, future studies should incorporate an instrument to assess coping behavior in more detail, such as the Coping Inventory for Stressful Situations (Endler and Parker, 1994) or the MND Coping Scale (Lee et al., 2001). Furthermore, our study used self-reporting instruments, which could be prone to biases, such as social-desirable responding. Another limitation of our study might be that patients with high achievement motives may be willing to participate in studies despite severe physical restrictions. Patients with high social support may be overrepresented, as a family member may support study inclusion. As a result, most studies including patients with ALS in advanced stages may report a high wellbeing despite severe physical function loss due to successful coping strategies of the participants (and sufficient social resources). Additionally, 18% of patients presented with mild cognitive impairments according to age- and education-adjusted cut-off scores for ECAS, which may interfere with wellbeing. However, none of the patients presented with dementia, and according to previous research, it can be expected that mild impairments do not reduce patient's general decision capacity (Böhm et al., 2016). Finally, we hereby present cross-sectional data, which provide a snapshot in time with no indication of dynamics in the course of the disease. It is not entirely clear yet whether coping behavior differs during the disease course (Jakobsson Larsson et al., 2016), and different coping strategies appear to have different effects on wellbeing during the disease course (Matuz et al., 2015). Future research needs to investigate longitudinal changes in wellbeing during the disease course. Additionally, in our study, none of the demographic factors played a role regarding wellbeing; however, there may be possible associations between wellbeing and other aspects of personality (Abdullahi et al., 2020; Anglim et al., 2020), cultural background (Ciećwierska et al., 2023a,b), or spirituality (Bernard et al., 2017; Ciećwierska et al., 2023b), which should be included in further research.

## 5 Conclusion

We present evidence from a large registry, indicating that patients with high self-attributed motives experience fewer coping difficulties in adapting to the changed circumstances associated with ALS, likely due to their intrinsic achievement motive. Additionally, anxiousness was closely related to fear of failure in general, while hope for success was associated with higher wellbeing. Active social participation showed a strong correlation with reduced depressiveness. These findings suggest that not only wellbeing but also coping traits and coping behavior should be monitored in patients dealing with ALS. This understanding about the coping process could be used for individual psychological counseling and intervention. Professional counseling and psychosocial intervention may improve adaptive coping behavior and wellbeing of patients (Kurt et al., 2007). Appropriate palliative care management can be enhanced by including regular social activities as a non-medicinal therapy, thereby preserving wellbeing even in the end-of-life stages of ALS.

## Data availability statement

The raw data supporting the conclusions of this article will be made available by the authors, without undue reservation.

## Ethics statement

The studies involving humans were approved by the Ethical Committees of University of Ulm (No. 11/10), the medical association of the state of “Baden-Wuerttemberg” (Landesärztekammer, No. B-F-2010-062), and the medical association of the state of “Bayern” (No. 7/11300). The studies were conducted in accordance with the local legislation and institutional requirements. The participants provided their written informed consent to participate in this study.

## Author contributions

JF: Conceptualization, Formal analysis, Methodology, Validation, Visualization, Writing – original draft, Writing – review & editing. AR: Conceptualization, Data curation, Writing – review & editing, Methodology. RP: Conceptualization, Methodology, Writing – review & editing. HB: Conceptualization, Data curation, Writing – review & editing. AB: Conceptualization, Data curation, Writing – review & editing. SD: Conceptualization, Data curation, Writing – review & editing. MS: Conceptualization, Data curation, Writing – review & editing. MH: Conceptualization, Data curation, Writing – review & editing. AK: Conceptualization, Data curation, Writing – review & editing. CO: Conceptualization, Data curation, Writing – review & editing. AN: Conceptualization, Data curation, Writing – review & editing. NS: Conceptualization, Data curation, Writing – review & editing. AL: Conceptualization, Data curation, Writing – review & editing. DR: Conceptualization, Methodology, Writing – review & editing. ACL: Conceptualization, Funding acquisition, Methodology, Project administration, Supervision, Writing – review & editing. GN: Project administration, Supervision, Writing – review & editing, Conceptualization, Methodology. DL: Conceptualization, Data curation, Formal analysis, Funding acquisition, Methodology, Project administration, Supervision, Writing – original draft, Writing – review & editing.

## The ALS Registry Study Group

Following cooperating partners provided data for the ALS Registry Swabia:

Andres F., Department of Neurology, Kreiskliniken Reutlingen; Arnold G., Department of Neurology, Klinikum Sindelfingen-Boeblingen; Asshauer I., Department of Psychiatry and Psychotherapy, Klinikum Friedrichshafen; Baier H., Department of Epileptology, ZFP Suedwuerttemberg; Beattie J., Department of Neurology, Ostalb-Klinikum Aalen; Becker T., Department of Psychiatry and Psychotherapy, BKH Guenzburg; Behne F., Department of Epileptology, ZFP Suedwuerttemberg; Bengel D., Department of Neurology, Oberschwabenklinik Ravensburg; Bracknies, V., Department of Neurology, Dietenbronn; Broer R., Department of Psychiatry and Psychotherapy, Weinsberg, Klinikum am Weissenhof; Burkhard, A., Department of Neurology, Klinikum Günzburg; Connemann B., Department of Psychiatry and Psychotherapy III, University of Ulm; Dettmers C., Schmieder Kliniken Konstanz; Dieterich M., Department of Neurology, LMU München; Etzersdorfer E., Department of Psychiatry and Psychotherapy, Furtbachkrankenhaus Stuttgart; Freund, W., Praxis Biberach; Gersner T., Department of Psychiatry and Psychotherapy, ZfP Zwiefalten; Gold H.-J., Department of Neurology, Klinikum am Gesundbrunnen Heilbronn; Hacke, W., Department of Neurology, University of Heidelberg; Haman G., Department of Neurology, Klinikum Günzburg; Heimbach B., Department of Neurology, University of Freiburg; Hemmer B., Department of Neurology, TU Muenchen; Hendrich C., Department of Neurology, Klinikum Friedrichshafen; Herting B., Department of Neurology, Diakonie-Klinikum Schwaebisch Hall; Huber R., Department of Neurology, Klinikum Friedrichshafen; Huber-Hartmann K., Department of Neurology, Kliniken Landkreis Heidenheim; Huelser P.-J., Department of Neurology, Fachklinik Wangen; Juettler E., Department of Neurology, Ostalb-Klinikum Aalen; Kammerer-Ciernioch J., Department of Psychiatry and Psychotherapy, Weinsberg, Klinikum am Weissenhof; Kaspar A., Department of Neurology, Oberschwabenklinik Ravensburg; Kern R., Department of Neurology, Klinikum Kempten; Kimmig H., Department of Neurology, Kliniken Schwenningen; Klebe, S., Department of Neurology, University of Würzburg; Kloetzsch C., Bodensee Klinikum, Schmieder Kliniken Allensbach; Klopstock, T., Department of Neurology, LMU München; Kuethmann A., Department of Psychiatry and Psychotherapy, Bezirkskrankenhaus Memmingen; Lewis D., Department of Neurology, Marienhospital Stuttgart; Lichy C., Department of Neurology, Klinikum Memmingen; Maeurer M., Department of Neurology, Caritas Krankenhaus Bad Mergentheim; Maier-Janson W., Praxis Ravensburg; Mertrikat J., Department of Neurology, Bundeswehrkrankenhaus Ulm; Meudt O., Department of Neurology, Klinikum Memmingen; Meyer A., Department of Neurology, Weissenau; Mueller vom Hagen J., Department of Neurology, Universitaetsklinikum Tuebingen; Naumann M., Department of Neurology and Neurophysiology, Klinikum Augsburg; Neher K.-D., Department of Neurology, Vinzenz von Paul Hospital, Rottweil; Neuhaus O., Department of Neurology, Kliniken Landkreis Sigmaringen; Neusch C., Praxis EMSA Singen; Niehaus L., Department of Neurology, Winnenden; Raape J., ZFP Suedwuerttemberg, Neurologie Weissenau; Ratzka P., Department of Neurology and Neurophysiology, Klinikum Augsburg; Rettenmayr C., Department of Neurology, Klinikum Esslingen; Riepe M. W., Department of Gerontopsychiatry, BKH Guenzburg; Rothmeier J., ZFP Suedwuerttemberg, Neurologie Weissenau; Sabolek M., Department of Neurology, Biberach; Schell C., Department of Neurology, Kreiskliniken Reutlingen; Schlipf T., Department of Psychiatry and Psychotherapy, Klinikum Schloss Winnenden; Schmauss M., Department of Psychiatry and Psychotherapy, Bezirkskrankenhaus Augsburg; Schoels L., Department of Neurology, Universitaetsklinikum Tuebingen; Schuetz K., Department of Neurology, Kliniken Schwenningen; Schweigert B., Department of Neurology, Caritas Krankenhaus, Bad Mergentheim; Sperber W., Department of Neurology, Kliniken Esslingen; Steber C., Department of Psychiatry and Psychotherapy, Bezirkskrankenhaus Augsburg; Steber R., Department of Psychiatry and Psychotherapy, Bezirkskrankenhaus Memmingen; Stroick M., Department of Neurology, Klinikum Memmingen; Trottenberg T., Department of Neurology, Winnenden; Tumani H., Department of Neurology, Dietenbronn; Wahl C., Department of Neurology, Klinikum Kempten; Weber F., Department of Neurology, Bundeswehrkrankenhaus Ulm; Weiler M., Department of Neuology, University of Heidelberg; Weiller C., Department of Neurology, University of Freiburg; Wessig C., Department of Neurology, University of Wuerzburg; Winkler A., Department of Neurology, TU Muenchen.
